# Unveiling Fatty Acid Profiles of the Parasitic Plants *Orobanche foetida* Poiret. and *Orobanche crenata* Forsk. and Modulation of Faba Bean (*Vicia faba* L.) Fatty Acid Composition in Response to Orobanche Infestation

**DOI:** 10.3390/plants12203578

**Published:** 2023-10-15

**Authors:** Amal Bouallegue, Siwar Thebti, Faouzi Horchani, Taoufik Hosni, Issam Nouairi, Haythem Mhadhbi, Najla Trabelsi, Moez Amri, Mohamed Kharrat, Zouhaier Abbes

**Affiliations:** 1 Laboratoire des Grandes Cultures, Institut National de la Recherche Agronomique de Tunisie (INRAT), University of Carthage, Rue Hédi Karray, Menzah 1004, Tunisia; siwarthebti2017@gmail.com (S.T.); taoufik.hosni@fsb.ucar.tn (T.H.); kharrat.mohamed@inrat.ucar.tn (M.K.); 2 Centre de Biotechnologie de Borj Cedria (CBBC), Laboratoire des Légumineuses et des Agrosys-Tèmes Durables, PB 901, Hammam Lif 2050, Tunisia; issam.nouairi@cbbc.rnrt.tn (I.N.); mhadhbihay@yahoo.fr (H.M.); 3 Laboratory of Biotechnology and Biomonitoring of the Environment and Oasis Ecosystems (LBBEEO), Faculty of Sciences of Gafsa, University of Gafsa, Zarroug, Gafsa 2112, Tunisia; faouzih20056@yahoo.fr; 4 Centre de Biotechnologie de Borj Cedria (CBBC), Laboratoire de Biotechnologie de l’Olivier, PB 901, Hammam Lif 2050, Tunisia; najlatrabe@yahoo.fr; 5 AgroBioSciences Program, College of Sustainable Agriculture and Environmental Science (SAES College), University Mohammed 6 Polytechnic (UM6P), Lot 660, Hay Moulay Rachid, Ben Guerir 43150, Morocco; moez.amri@um6p.ma

**Keywords:** chlorophyll, faba bean, fatty acids, lipid peroxidation, orobanche

## Abstract

Broomrapes (*Orobanche* spp.) are root parasitic plants that threaten agricultural production in many parts of the world. In this study, the effect of two orobanche species, *Orobanche crenata* and *O. foetida*, on faba bean plants was studied in Tunisia. The two orobanche species inhibited both biomass production and pod formation, decreased the chlorophyll (Chl) content and total lipid (TL), and enhanced electrolyte leakage (EL) and lipid peroxidation. Concomitantly, orobanche parasitism induced a lower degree of fatty acid (FA) unsaturation due to a shift in the FA composition. On the other hand, with regard to orobanche seeds, oleic and linoleic acids were the predominant FA in the two orobanche species. After orobanche seed germination and penetration of host tissues, all the orobanche development stages showed a decrease in the TL content and changes in the FA composition in comparison to orobanche seeds. The level of TL was equal to or lower in all parasite development stages (except for S4) than that in the roots and leaves of healthy faba bean plants. These results suggest that the negative effect of orobanche infestation on faba bean development can be attributed to the reduced chlorophyll content and alteration in membrane stability attested by the reduced TL level and FA unsaturation.

## 1. Introduction

Broomrapes are parasitic flowering plants that cause important damage in many cultivated crop species. These parasites are entirely dependent on their hosts for their nutritional requirements. *Orobanche crenata*, *O. cumana*, *Phelipanche aegyptiaca*, *P. ramosa*, and *O. minor* are the most harmful species [1]. In Tunisia, *O. crenata* and *O. foetida* are considered the major parasitic weeds, and faba bean yield losses caused by *O. foetida* are estimated to be 66–90% in the Beja region (Tunisia) [2].

The germination of orobanche seeds, induced by strigolactones in root exudates of host plants, results in the formation of a haustorium, which serves as a bridge for the transfer of nutrients from the host phloem to the parasitic plant. After orobanche attachment on roots, a subterranean tubercle is developed, giving rise to a flowering spike after emergence from the soil [1,3].

Osmoregulation and nutritional relationships between hosts and parasites have been studied by several authors [4,5,6]. These studies showed that osmolarity was higher in tubercles than in the host root, facilitating nutrient transfer from the host to the parasitic plant. For the case of the system faba bean (cv. Bachaar)/*O. foetida*, Abbes et al. [5,6] showed that carbohydrates (such as sucrose and stachyose), organic acids, and amino acids were the major organic components transferred from the host to the parasite. However, hexoses and soluble amino acids, in particular, were preferentially accumulated by the parasite. The accumulation of hexoses was also detected in *O. crenata* [4,7], *O. aegyptiaca* [8], *O. hederae* [9], and *P. ramosa* [10]. The implication of invertases and glutamine-dependent asparagine synthetase enzymes in the C and N metabolism of the parasite could explain the difference in the organic components accumulated by the host and parasite [6]. Abbes et al. [5] indicated that the phloem composition of the susceptible cv. Bachaar did not change significantly in response to the *O. foetida* attack.

On the other hand, studies on lipid peroxidation, membrane permeability, and fatty acid (FA) profile in plants infested by orobanche species are scarce. The FAs are important molecules that play multiple crucial roles in plants. They have structural functions as constituents of phospholipids, which are the building blocks of cell membranes [11]. They also serve as biologically active molecules that can be involved as signal transduction mediators [12] and as energy sources for various metabolic processes [13]. In addition, they are implicated directly and indirectly in stress defense via multiple mechanisms [14]. Very few studies have investigated the effect of plant parasite infestation on the lipid content and the FA composition of hosts. Only a small number of studies have explored the effect of *Cuscuta* parasitism on hosts [15,16,17]. The present study deals with the lipid composition of two orobanche species, *O. crenata* and *O. foetida*, and investigates in parallel alteration in the levels of TL content and FA composition after the infestation of faba bean plants by these two plant parasites.

## 2. Results

### 2.1. Effect of Orobanche Infestation on Faba Bean Development

Broomrape infestation significantly decreased faba bean root and shoot dry weight (DW) by 61.51% and 47.66% for *O. crenata* and by 83.02% and 67.26% for *O. foetida*, respectively. No pod formation was detected for infested plants with the two orobanche species. In contrast, the shoot length was not affected by the broomrape infestation (Table 1).

The orobanche attachment number and DW did not differ significantly between the two orobanche species, with around 30% of tubercles evolving into emerged spikes (S5). No significant differences were observed in subterranean attachments and emerged spikes between the two orobanche species (Table 2).

### 2.2. Chlorophyll Contents

**Figure 1:**
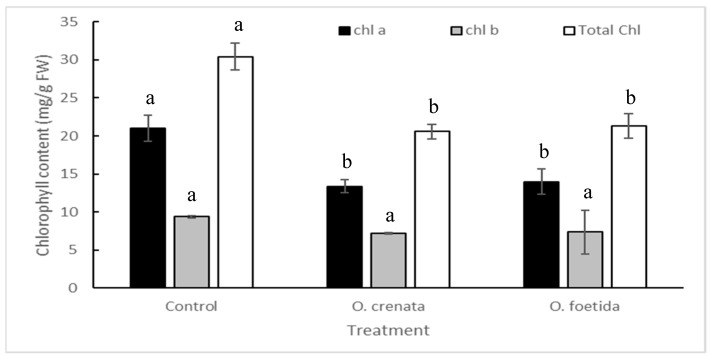
Chlorophyll content (mg/g FW) in leaves of non-infested and orobanche-infested cv. Bachaar plants. Data are means ± SE. For each parameter, data with different letters are significantly different (*p* = 0.05, Duncan test), n = 3.

The chl a content was predominant and showed significant differences between non-infested and infested plants (Figure 1). However, for the chl b content, no significant differences were observed. Parasitism decreased the total chlorophyll content by 32.32% and 29.82% with *O. crenata* and *O. foetida*, respectively.

### 2.3. Malondialdehyde (MDA) Content and Electrolyte Leakage (EL)

**Figure 2:**
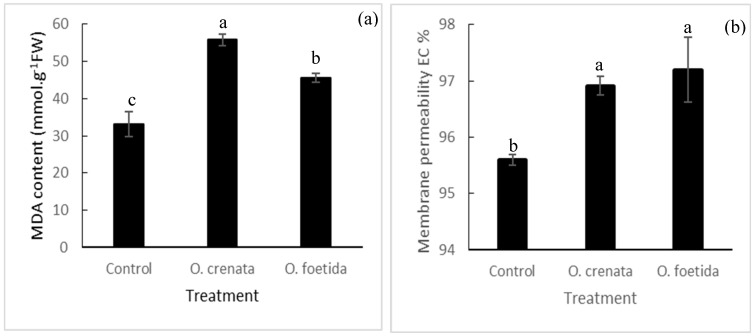
Effect of orobanche infestation on the MDA content (**a**) and EL (**b**) in leaves of cv. Bachaar plants. Data are means ± SE. Data with different letters are significantly different (*p* = 0.05, Duncan test), n =3.

There was an increase in the MDA content and EL in response to the orobanche infestation as compared with the control treatment (Figure 2). The MDA content and electrolyte leakage increased by 68.41% and 1.31% with *O. crenata* and by 37.54% and 1.60% with *O. foetida*, respectively, in comparison with the control treatment.

### 2.4. Total Lipid Content and Fatty Acid Composition in cv. Bachaar Plants

The parasitic impact of both orobanche species on faba bean plants significantly decreased the TL content of host leaves. This decrease was 43.64% and 56.36% upon infestation by *O. crenata* and *O. foetida*, respectively. However, no significant decrease in the TL content in host roots in response to both orobanche species was observed (Table 3).

Table 4 shows that in tissues of non-infested plant leaves, linolenic acid was the predominant FA. However, the predominant FAs were palmitic and stearic acids upon *O. crenata* infestation and palmitic, stearic, and linolenic acids upon *O. foetida* infestation. In roots, palmitic and stearic FAs were the predominant FAs in both non-infested and infested plants.

In leaves, the parasitism by *O. crenata* significantly increased the palmitic and stearic acids and decreased oleic, linoleic, and linolenic acid percentages. However, the alteration in the other FAs was insignificant. *O. foetida* induced a significant increase in the percentage of stearic acid and a decrease in oleic and linoleic acids. However, the alterations in palmitic, linolenic, and other FAs were insignificant. In roots, the two orobanche species increased the palmitic acid and decreased the oleic acid percentages. The percentages of the other FAs did not change significantly after the orobanche infestation (Table 4). The percentage of unsaturated FA of faba bean leaves decreased by 43.77% and 20.78% upon infestation by *O. crenata* and *O. foetida*, respectively. In roots, this percentage did not show significant changes (Table 4).

### 2.5. Total Lipid Content and Fatty Acid Composition in Parasitic Plants

The two orobanche species were characterized by similar levels of TL content and FA composition (Table 5 and Table 6). For the two orobanche species, the highest amount of TL was observed in seeds, with more TL content in *O. foetida* than in *O. crenata*. The other development stages (S2–S5) showed a significant decrease in the TL content in comparison with seeds. The difference between stages (S2–S5) was not significant.

The FA composition of the TL content in dry seeds and subsequent development stages is presented in Table 6. The predominant FAs were oleic and linoleic acids in the two orobanche seeds, accounting for 49.83% and 31.18% in *O. crenata* and 54.36% and 28.99% in *O. foetida*, respectively. The other FAs ranged from 0.24% to 10.27%.

The FA percentages were changed after orobanche seed germination and penetration in host roots. The two orobanche species and their development stages were characterized by similar FA compositions. For the two orobanche species, during the first development stages (stages S2 + S3) and the final stage of development (Stage S5), the predominant FAs were palmitic and linoleic acids. However, during stage S4, palmitic and stearic acids were the predominant FAs, with 34.04% and 40.00% for *O. crenata* and 33.08% and 30.71% for *O. foetida*, respectively (Table 6). The percentage of unsaturated FAs decreased significantly in all development stages (S2–S5) in comparison to seeds of the two orobanche species.

## 3. Discussion

Broomrapes are angiospermic root parasites that cause morphological and metabolic perturbation in susceptible hosts. In this study, the orobanche infestation reduced root and shoot dry weight and no pod production was observed on infested cv. Bachaar plants. The susceptible cv. Bachaar showed similar behaviors in response to infestation by the two orobanche species. Similar observations were reported in other studies [18,19].

The reduction in pod setting in cv. Bachaar can be explained by the alteration of photosynthetically active pigments. Our results showed that the two orobanche species lead to a decrease in the chl content, especially chl a. The effect of orobanche parasitism on the chl content was also observed on other host–parasite relations, such as chickpea-*O. foetida*, faba bean-*O. foetida*, and tomato-*P. ramosa* [18,19,20,21].

In the present work, the effect of orobanche infestation on membrane integrity could be related to changes in TL content and FA profiles (Table 3 and Table 5). *O. crenata* and *O. foetida* infestation resulted in a decrease in the TL content of faba bean plants, especially in leaves. Similar observations were made by Mostafa et al. [22] and Abbes et al. [3], who found decreased lipid content in orobanche-infested faba bean leaves. Mishra & Sanwal [15] also observed a decrease in the lipid content of *Brassica juncea* seeds following infestation by *Cuscuta reflaxa*. On the contrary, Sharma et al. [17] mentioned the higher TL content in several host plants infested with *C. reflaxa*. The reduced lipid levels in orobanche-infested Bachaar plants may be related to higher lipid degradation. In fact, results showed that lipid peroxidation and EL levels significantly increased in response to orobanche infestation in comparison with the control (Figure 2). The stimulation effect of broomrapes on MDA (a major product of lipid peroxidation) was reported in several studies [3,23]. The current results indicated that *O. crenata* and *O. foetida* infestation caused oxidative stress by disturbing redox homeostasis and accumulating reactive oxygen species (ROS), probably through increasing the photorespiration rate and NADPH oxidase activity in host plants. Consequently, these could impair cell macromolecules through oxidation of DNA, lipids, and proteins and eventually decrease cell viability [24]. These results were consistent with the findings of Dos Santos et al. [25], who found that various genes involved in oxidative stress were up-regulated in *Arabidopsis thaliana* plants after a few hours of infection with *O. ramosa*. Additionally, *O. cumana*-infested sunflower and *A. thaliana* exhibited a significant accumulation of H_2_O_2_ contents as compared with uninfected plants [26]. On the other hand, Mishra & Sanwal [16] showed that the infestation of rape plant by *Cuscuta* resulted in a lipid reduction in leaf chloroplast, probably associated with high lipase activity in host leaves.

Faba bean leaves showed that the predominant FA was linolenic acid. Similarly, Arbaoui & Link [27], Ryu et al. [28], and Abbes et al. [3] reported that linolenic acid is the main FA in faba bean leaves. However, immature pods and seeds showed linoleic acid as the predominant FA [28]. In general, the two orobanche species induced a decrease in unsaturated FA and an increase in saturated ones. Our results showed that the percentage of unsaturated FA of infested faba bean plants decreased, especially in leaves (Table 4). This is in accordance with the results of Mishra & Sanwal [15], who showed a decrease of 23% in the percentage of unsaturated FA in *B. juncea* infested by *Cuscuta*. Similarly, Abbes et al. [3] indicated that the orobanche parasitism decreased the unsaturated FA percentage in leaves of the susceptible cv. Badi (up to 41%), but not in resistant varieties Najeh and Chourouk. The decrease in the tri-unsaturated FA observed under the orobanche infestation probably led to reduced fluidity and permeability of lipid membranes.

In the two parasitic plants, TL content was equal to or lower in all parasite development stages (except S4) than in the roots and leaves of healthy faba bean plants (Table 3 and Table 5). This confirms the results obtained by Mostafa et al. [22], who studied the relation of *V. faba* with *O. crenata*. In the case of *Cuscuta reflexa*, this level was higher in the parasite than in its hosts [17]. The authors indicated that the lipid level of *C. reflexa* was related to the lipid content of the host plants. In the present study, oleic and linoleic acids were the predominant FAs in seeds of the two orobanche species (Table 6). Similar results were observed by Velasco et al. [29] and by Bar-Nun & Mayer [30], who studied the FA composition in *P. aegyptiaca* seeds. With respect to *O. cernua* and *O. cumana*, Pujadas-Salva & Velasco [31] reported that *O. cernua* is characterized by high oleic acid concentration. However, *O. cumana* showed a high linoleic acid concentration. Velasco et al. [29] examined the ratio of oleic to linoleic acids in a number of species of Orobanche from different accessions. They showed that in most cases, the ratio was two or less. In our case, this ratio was 1.60 for *O. crenata* and 1.87 for *O. foetida* seeds. In general, several factors could explain the differences observed in the FA profiles of the different orobanche species, such as the plant lipid metabolism, the harvest location, and the diversity of metabolic precursors derived from different host plants.

Seeds of the two orobanche species showed higher amounts of lipids. Similarly, Thomas et al. [32] and Aber & Sallé [33] reported that orobanche seeds are rich in lipid reserves. Bar-Nun and Mayer [29] indicated that during orobanche seed conditioning, significant changes were observed in carbohydrate metabolism and protein synthesis but not in nitrogen lipid contents. According to Joel & Losner-Goshen [34], both the endosperm and embryo of *O. cumana* and *P. aegyptiaca* seeds showed lipids as the main storage material.

In our results, after orobanche germination and penetration of host tissues, all the orobanche development stages (S2–S5) showed changes in the TL content and FA composition in comparison with orobanche seeds. The TL content decreased significantly, and the predominant FA also changed. The percentage of unsaturation decreased significantly in all development stages (S2–S5) of the two orobanche species. An increase in the percentage of saturated FA was observed, especially in the development stage S4. These changes are in accordance with studies of Velasco et al. [29], who showed high levels of oleic, linoleic, and palmitic acids in orobanche seeds, and Ben Attia et al. [35], who showed, in contrast, that the lipids extracted from the whole plants (flowering stage) of the parasitic weeds *Cistanche violacea*, *O. crenata*, and *O. lavandulacea* predominantly contained linoleic and palmitic acids. According to Chapman et al. [36], the pathways of lipid synthesis may vary depending on the parasitic development stage.

The decrease in the TL content in the attached parasites (Table 5) and the increases in reducing sugars, sucrose, and starch [5,6] can be explained by the conversion of FA into carbohydrates through the glyoxylate cycle. Joel & Losner-Goshen [34] found that the orobanche embryonic phase is characterized by a large accumulation of lipids, while the parasitic phase (developed parasitic plants) instead showed an accumulation of starch. Several studies have mentioned the presence of large quantities of starches in different mature orobanche shoots [5,6,8].

Overall, these results showed the negative effects of the orobanche infestation on faba bean development and pod setting and production. This can be attributed to the reduced chlorophyll content and increased accumulation of MDA and EL levels, resulting in the alteration of membrane stability attested by the reduced TL level and FA unsaturation. Such findings could be useful in future research activities targeting the development of a resistant faba bean germplasm through the combination of different resistance mechanisms.

## 4. Materials and Methods

### 4.1. Plant Culture

The commercial faba bean cv. Bachaar was used in this study. This variety is known for its susceptibility in *O. crenata*- and *O. foetida*-infested fields [2,19]. *O. crenata* and *O. foetida* seeds were collected in 2015 from mature parasitic plants in infested faba bean fields, respectively, from Ariana and Beja (Tunisia).

Faba bean and orobanche seeds were sterilized in calcium hypochlorite (1%). The faba bean plants were grown in 10 l pots containing a substrate of sterilized soil, non-infested or artificially infested with 20 mg of *O. crenata* or *O. foetida* seeds per kg. Five pots were prepared for each treatment. The experiment was conducted in a greenhouse at 20 ± 3°C, 70% relative humidity, and 16 h photo-period. Plants were watered as needed. Four months after planting (at maturity stage), roots of non-infested and infested plants were removed from the substrate, and the orobanche attachments were harvested and classified into S1–S5 stages with S1: haustorium attached to host root; S2: small orobanche tubercles without root formation; S3: orobanche tubercles without shoot formation; S4: underground orobanche tubercles with shoot development; and S5: emerged spikes [37]. SH, SDW, RDW, and orobanche number and DW per plant were also determined. DW was determined after drying the fresh samples at 80 °C for 48 h. Host and parasite tissue samples were also collected for physiological and biochemical analyses.

### 4.2. Determination of Lipid Peroxidation

In this experiment, a 0.5 g tissue sample was homogenized in 1 mL 5% trichloroacetic acid (TCA) and then centrifuged at 10,000× *g* for 15 min. Then, 1 mL of 20% TCA containing 0.5% 2-thiobarbituric acid (TBA) was added to the supernatant (1 mL) and heated at 95 °C for 15 min. The non-specific absorbance of cooled extracts was measured at 600 nm and subtracted from the specific absorbance readings (532 nm). Results were expressed as μmoles of malondialdehyde (MDA)/g fresh weight formed using an extinction coefficient of 155 mM/cm [38].

### 4.3. Determination of Membrane Permeability

Tissue samples (100 mg) were cut into uniform discs, placed in tubes containing 10 mL of deionized water, and vibrated for 30 min, and the first medium conductivity (EC1) was determined. Then, the samples were boiled for 15 min, and EC2 (final conductivity) was measured. The EL percentage was determined using the following the formula: (EC1/EC2) × 100.

### 4.4. Determination of Chlorophyll Contents

Chlorophyll (chl a, chl b, chl t) contents were determined as described by Arnon [39]. Measurements were made at the pod setting stage on leaves (from the fifth nodes) of control and infested plants.

### 4.5. Lipid Extraction and Determination of FA Composition

Tissue samples were boiled in water for 5 min, homogenized in chloroform: methanol (1:1, *v*/*v*), and then centrifuged at 3000× *g* for 15 min (Neofuge 13R model). Lipids (chloroformic phase) were aspired and evaporated under vacuum using a rotary evaporator. The residue was redissolved in 2 mL toluene: ethanol mixture (4:1, *v*/*v*) for conservation [40]. FAs were methylated using the method of Metcalfe et al. [41]. Methyl esters of total FA were separated and quantified using gas chromatography (GC) (HP 4890 D, Hewlett-Packard Company, Wilmington, DE, USA) equipped with an Innowax capillary column (30 m × 0.53 mm × 0.25 µm), which was maintained isothermally at 210 °C. The detector was a flame ionization detector (FID). For measuring the amounts of fatty acids, heptadecanoic acid (17:0) was added as an internal standard. Calculation of FA quantities was performed using an integrator HP model 3390 A.

### 4.6. Statistical Analysis

The statistical analyses were conducted using SPSS software (Version 19.0 for Windows). ANOVA was performed employing a general linear model, with treatments considered as fixed factors. All measurements were carried out in triplicate. Significance levels were set at *p* = 0.05, and Duncan’s multiple-range test was employed for pairwise comparisons.

## Figures and Tables

**Table 1:** Shoot height (SH, cm), shoot DW (SDW, g), root DW (RDW, g), and pod number (PN) in non-infested and orobanche-infested cv. Bachaar plants.

	SH (cm)	SDW (g)	RDW (g)	PN
Control	69.60 a ^a^	4.49 a	2.65 a	2.00 a
*O. crenata*	58.00 a	2.35 b	1.02 b	0.00 b
*O. foetida*	58.80 a	1.47 c	0.45 c	0.00 b

^a^ Data with different letters within a column are significantly different (*p* = 0.05, Duncan test), n = 3.

**Table 2:** Total orobanche number and dry weight (g) attached to cv. Bachaar plants.

	Total Orobanche Number	S2 ^b^	S3	S4	S5	Non-Emerged Orobanche Number	Orobanche DW (g)	% S5
*O. crenata*	21.20 a ^a^	0.00 a	0.80 a	14.20 a	6.20 a	15.00 a	2.51 a	29.9 a
*O. foetida*	18.80 a	0.60 a	2.40 a	10.20 a	5.60 a	13.20 a	3.19 a	29.2 a

^a^ Data with different letters within a column are significantly different (*p* = 0.05, Duncan test), n = 3. ^b^ S2: small orobanche tubercles without root formation; S3: orobanche tubercles without shoot formation; S4: underground orobanche tubercles with shoot development; S5: emerged spikes.

**Table 3:** Total lipid content (mg/g FW) in roots and leaves of non-infested and orobanche-infested cv. Bachaar plants.

	Bachaar Leaves	Bachaar Roots
Control	3.30 a ^a^	2.72 a
*O. crenata*	1.86 b	2.03 a
*O. foetida*	1.44 b	1.83 a

^a^ Data with different letters within a column are significantly different (*p* = 0.05, Duncan test), n = 3.

**Table 4:** Fatty acid composition (% of total lipids) in roots and leaves of non-infested and orobanche-infested cv. Bachaar plants.

Organ	Treatment	C16:0 ^b^	C18:0	C18:1	C18:2	C18:3	Others	Unsaturation
Leaves	Control	18.09 b ^a^	10.44 c	15.14 a	11.92 a	37.60 a	6.81 a	65.36 a
	*O. crenata*	31.57 a	39.74 a	3.56 c	3.53 c	14.17 b	7.44 a	21.59 c
	*O. foetida*	21.83 b	26.71 b	8.08 b	8.12 b	28.11 a	7.13 a	44.58 b
Roots	Control	32.71 c	47.08 a	5.09 a	5.04 a	2.71 a	7.37 b	12.99 a
	*O. crenata*	35.04 b	44.71 a	3.48 b	6.58 a	3.00 a	7.19 b	13.36 a
	*O. foetida*	37.06 a	41.13 a	3.09 b	7.33 a	2.25 a	9.13 a	14.08 a

^a^ For each organ, data with different letters within a column are significantly different (*p* = 0.05, Duncan test), n = 3. ^b^ C16:0: palmitic acid, C18:0: stearic acid, C18:1: oleic acid, C18:2: linoleic acid, C18:3: linolenic acid, and “Others” include minor fatty acids such as C12:0: lauric acid, C14:0: myristic acid, C16:1: palmilotic acid, C20:0: arachidic acid, and C22:0: behenic acid.

**Table 5:** Changes in TL contents (mg/g FW) in *O. crenata* and *O. foetida* seeds and subsequent development stages of cv. Bachaar plants.

	*O. crenata*	*O. foetida*
Seeds	19.40 a ^a^	33.94 a
Tubercles S2 + S3 ^b^	0.91 b	1.29 b
Tubercles S4	4.21 b	4.66 b
Orobanche S5	1.41 b	1.27 b

^a^ Data with different letters within a column are significantly different (*p* = 0.05, Duncan test), n = 3. ^b^ See Table 2.

**Table 6:** Changes in fatty acid composition (% of total lipids) in *O. crenata* and *O. foetida* seeds and subsequent development stages of cv. Bachaar plants.

Orobanche Species	Development Stage	C16:0 ^b^	C18:0	C18:1	C18:2	C18:3	Others	Unsaturation
*O. crenata*	Seeds	10.27 d ^a^	6.16 c	49.83 a	31.18 b	0.64 b	1.91 b	81.76 a
	Tubercles S2 + S3 ^c^	27.68 b	14.08 b	6.64 b	29.14 b	11.45 a	11.00 a	49.19 b
	Tubercles S4	34.04 a	40.00 a	3.53 b	10.76 c	2.04 b	9.64 a	16.45 c
	Orobanche S5	23.35 c	14.77 b	6.40 b	41.03 a	9.54 a	4.91 b	57.51 b
*O. foetida*	Seeds	9.68 c	4.50 b	54.36 a	28.99 b	0.24 d	2.22 c	83.72 a
	Tubercles S2 + S3	26.10 b	7.69 b	3.54 b	41.68 a	17.46 a	3.52 bc	63.33 b
	Tubercles S4	33.08 a	30.71 a	2.53 b	18.76 c	7.76 c	7.16 a	29.13 c
	Orobanche S5	25.64 b	6.80 b	5.02 b	43.04 a	14.82 b	4.68 b	63.31 b

^a^ For each orobanche species, data with different letters within a column are significantly different (*p* = 0.05, Duncan test), n = 3. ^b^ See Table 4. ^c^ See Table 2.

## Data Availability

The data presented in this study are available on request from the corresponding author.
